# The effect of the scale of grant scoring on ranking accuracy

**DOI:** 10.12688/f1000research.125400.1

**Published:** 2022-10-19

**Authors:** Peter M. Visscher, Loic Yengo

**Affiliations:** 1 Institute for Molecular Bioscience, The University of Queensland, St Lucia, Queensland, 4072, Australia

**Keywords:** grant scoring, multiple threshold model, grant quality, grant ranking

## Abstract

In this study we quantify the accuracy of scoring the quality of research grants using a finite set of distinct categories (1, 2, …., k), when the unobserved grant score is a continuous random variable comprising a true quality score and measurement error, both normally distributed. We vary the number of categories, the number of assessors that score the same grant and a signal-to-noise ratio parameter. We show that the loss of information of scoring a small number of categories (k > 5) compared to scoring on a continuous scale is very small, so that increasing the number of scoring categories is unlikely to lead to an improvement in the outcomes of scoring systems. In addition, we model the effect of grant assessors scoring too close to the mean and show that this results in only a very small reduction in the accuracy of scoring.

## Introduction

Grant funding bodies use different ways to obtain a final ranking of grant proposals. The number of items that are scored can vary, as well as the scale on which each item is scored and the weighting scheme to combine the individual items scores into a single overall score. This note only concerns the matter of the scale of scoring.

Scoring scales differ widely among grant funding bodies. For example, in Australia, the National Health and Medical Research Council (NHMRC) uses a scale of 1-7 whereas the Australian Research Council (ARC) uses 1-5 (A:E), and other funding bodies use scales such as 1-10. One question for the funding bodies, grant applicants and grant assessors is whether using a different scale would lead to more accurate outcomes. For example, if the NHMRC would allow half-scores (e.g., 5.5), expanding the scale to 13 categories (1, 1.5, …, 6.5, 7), or the ARC would expand to 1-10, then might that lead to a better ranking of grants? This is the question we address in this note. Specially, we address two questions that are relevant for grant scoring: (1) how much information is lost when scoring in discrete categories compared to scoring on a scale that is continuous; and (2) what is the effect of the scale of scoring on the accuracy of the ranking of grants?

## Methods

To quantify the effect of grant scoring scale on scoring accuracy, a model of the unknown true distribution of grant quality has to be assumed, as well as the distribution of errors in scoring the quality of a grant. We assume a simple model where an *unobserved* underlying score ( *u*) is continuous (so no discrete categories) and the error ( *e*) is randomly distributed around the true quality ( *q*) of the grant and that there is no correlation between the true quality of the grant and the error,

$$u_{i}=q_{i}+e_{i},$$

with *u _i_* the score of grant *i* on the underlying continuous scale, *q _i_* its quality value on that scale and *e _i_* a random deviation (error). Furthermore, we assume that *q* and *e* are normally distributed around zero and, without losing generality, that the variance of *u* is 1. Hence, σ ^2^_u_ = σ ^2^_q_ + σ ^2^_e_ = 1. We denote the signal-to-noise ratio as s = σ ^2^_q_/ (σ ^2^_q_ + σ ^2^_e_), which is a value between zero and one. Note that adding a mean to model and/or changing the total variance of *u* will not change subsequent results. This continuous scale is never observed unless the scoring system would allow full continuous scoring. A close approximation of this scale would be if the scoring scale were to be continuous in the range of, for example, 1-100. In summary, we propose a simple signal ( *q*) + noise ( *e*) model on an underlying scale which is continuous.

We now define the way in which the grants are actually scored by assessors. Assume that there are k mutually exclusive categories (e.g., k = 7) which correspond to (k-1) fixed thresholds on the underlying scale and k discontinuities on the observed ( *Y*) scale. We also assume that the scores on the *Y*-scale are linear and symmetrically distributed, so for an even number of categories, there will be a threshold on the *u*-scale located at zero (formally, if there are k categories then threshold t _k/2_ = 0). This is an example of a multiple threshold model. In the extreme case of k = 2 (assessors can only score 1 or 2), the threshold on the underlying *u*-scale is 0 and when *u* < 0 then the observed score *Y* = 1 and when *u* > 0 then the observed score *Y* = 2. The mean on the observed scale in this model is simply (k + 1)/2.

**Figure 1.**
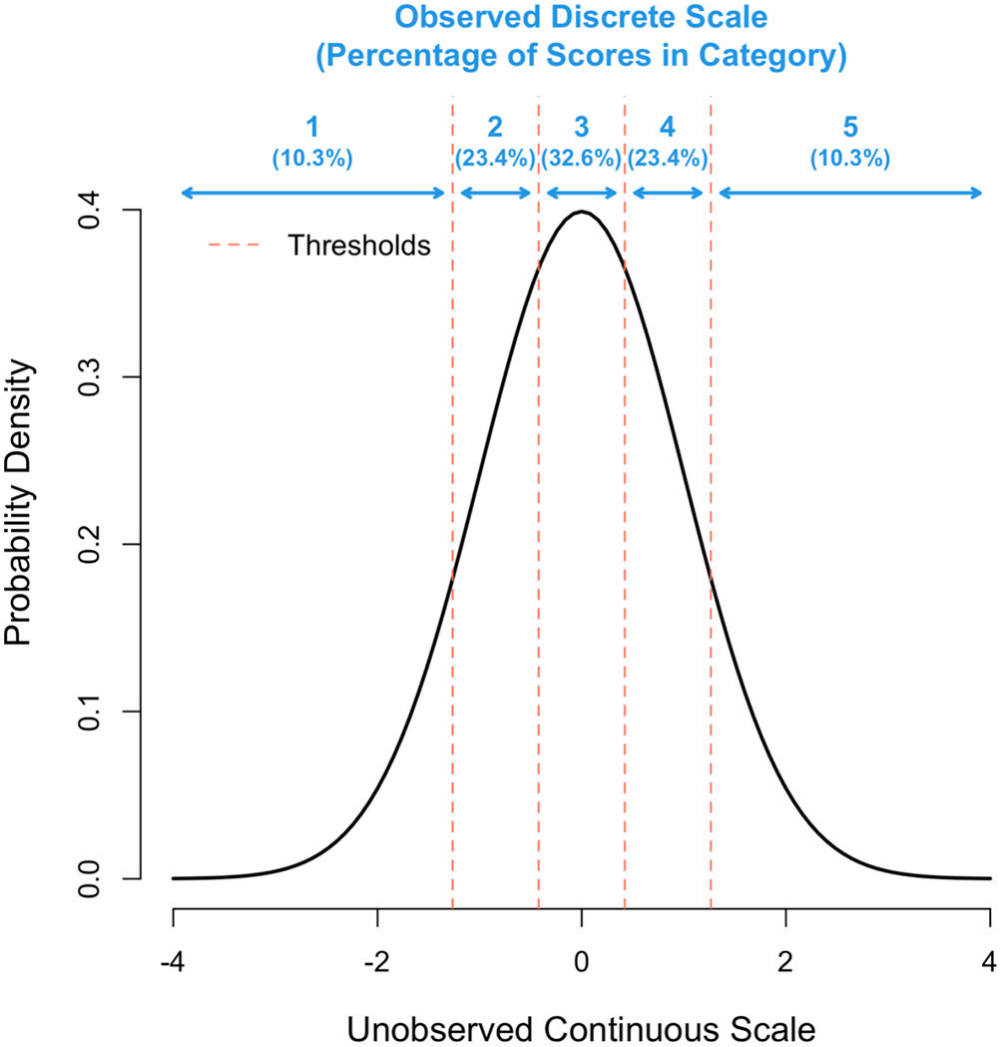
Representation of the multiple threshold model for k = 5 categories. The x-axis shows the unobserved continuous scale in standard deviation units and the y-axis the density. The position of each of the 4 thresholds is shown as a vertical red line.

In summary, we assume that the actual observed score is a response in one of several mutually exclusive categories (1, 2, …, k), which arise from an unobserved underlying continuous scale. For a given number of categories (k), the (k-1) thresholds were determined to maximise the correlation between the observed Y-scale and the unobserved continuous u-scale, while fixing the inter-threshold spacing on the u-scale to be constant. This ensures symmetry on the observed Y-scale and appears to be the optimal solution in that it gave identical results to a general optimisation of the thresholds (results not shown). Figure 1 gives a schematic for k = 5. To get the thresholds requires a numerical optimisation, which was done through a purpose-written program using the statistical software package R version 4.2.0 (see Software availability section). The question on the loss of information by using a finite versus continuous (infinite) scoring scale was addressed by calculating the correlation between *Y* (observed) and u (continuous) with increasing values of k from 1 to 100. For a given set of thresholds t _i_ and assuming that the variance on the underlying scale ( *u*) is 1, this correlation (R _k_) was calculated as,

$$R_{k}=\text{Corr}\left(Y,,,u\right)=\left[\sum \;\left(z_{i}\right)\right]/\surd \left(\mathrm{var}\left(Y\right)\right)$$ [1]

with *z _i_* (i = 1, … k-1) the height of the normal curve pertaining to threshold *t _i_*, and var( *Y*) the variance of the observed scores, which is calculated from the proportions *p _i_* (i = 1, …,k) of scores that fall into category *i*, which in turn follow from the thresholds *t _i_.*

$$\mathrm{var}\left(Y\right)=\sum \;\left(p_{i}{Y_{i}}^{2}\right)–{\left(\sum p_{i}Y_{i}\right)}^{2}$$ [2]

The expression for the correlation between the observed and underlying scale under the multiple threshold model is known from the genetics literature ( Gianola, 1979). The square of the correlation in Equation [1] is the proportion of variation on the continuous scale that is captured by the discrete scale. For k = 2, *t*_1_ = 0, *z*_1_ = 0.3989, *p*_1_ = *p*_2_ = ½, *Y*_1_ = 1 and *Y*_2_ = 2, giving var( *Y*) = ¼ and R _2_ ~ √0.637 = 0.798. This is a known result for a threshold model with two equal categories, where the binary scale captures 63.7% of the variation on the continuous scale ( Dempster and Lerner, 1950).

To address the question on the effect of scoring on the ranking of grants we need to estimate the signal-to-noise ratio of the Y-scale and u-scale. Thresholds models with two random effects on the underlying scale have been studied in the genetic literature (e.g., Dempster and Lerner, 1950; Gianola, 1979; Gianola and Norton, 1981). Gianola (1979) also deals with the case where the errors ( *e*) are exponentially distributed, but this distribution was not considered here.

When the observed scores are 1, 2, …, k, Gianola (1979) showed that the ratio of signal-to-noise on the observed Y-scale and unobserved u-scale is R _k_^2^, the square of the correlation in Equation [1]. Therefore, the ratio of signal-to-noise parameters (R _k_^2^) does not depend on the signal-to-noise value on the underlying scale (s) itself. However, the effect of scaling on the ranking of grants does depend on the signal-to-ratio effects, and to address this question we need to also specify the number of assessors (m). Given m (e.g., m = 4, 5, 6), the correlation ( *Corr*) between the true score of a grant ( *q _i_*) and the mean score from m assessors on the *u*-scale or *Y*-scale can be shown to be,

$${\text{Corr}}_{u}=\surd \left[m/\left(m+\lambda_{u}\right)\right],\text{with }\lambda_{u}={\sigma^{2}}_{e}/{\sigma^{2}}_{q}=\left(1-s\right)/s$$

$${\text{Corr}}_{Y}=\surd \left[m/\left(m+\lambda_{Y}\right)\right],\text{with }\lambda_{Y}=\left(1-{R_{k}}^{2}s\right)/\left({R_{k}}^{2}s\right)$$

Finally, we can express the loss of information in ranking grants when m assessor score on the *Y*-scale instead of on the continuous scale as,

$$L\left(m,k,s\right)=1-{\text{Corr}}_{Y}/{\text{Corr}}_{u}=1-\surd \left[\left(m+\lambda_{u}\right)/\left(m+\lambda_{Y}\right)\right]$$ [3]

Equations [1] and [3] can also be used to compare different values for k against each other. For example k = 7 versus k = 13 can be compared by calculating R _7_/R _13_ and L(m,13,s)/L(m,7,s).

Grant assessors might not use the entire scale that is available to them or score too few grants in the extreme categories (categories 1 and k, respectively). The effect of such a scoring approach is to change the proportions in each of the k categories and thereby change the variance on the Y-scale and the covariance between the *u* and *Y* variables. These changes lead to a lower correlation between *Y* and *u* than given by Equation [1] and, consequently, reduce the ranking accuracy of grants. We simulated this scenario by using the same model as before, but now assuming that the proportions of scores in each category follow from a normal distribution with smaller variance (σ ^2^_us_) than the variance of 1 which is assumed to be the true unobserved variance (when σ ^2^_us_ = σ ^2^_u_ = 1). When σ ^2^_us_ < 1, this model leads to more scores around the mean and fewer in the tails (the lowest and highest category).

## Results

**Figure 2.**
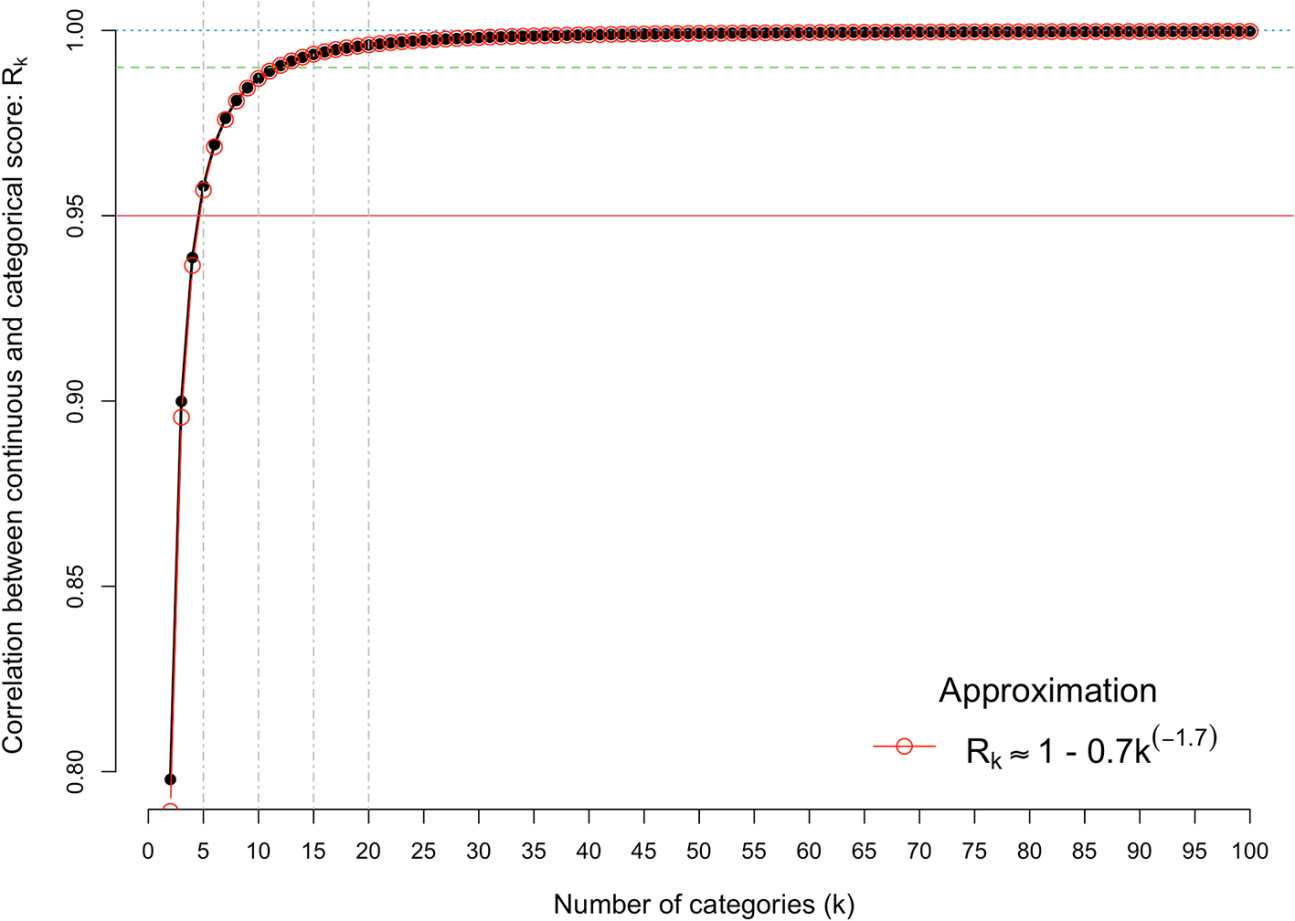
Correlation between the observed categorical score and the underlying continuous score. The x-axis is the number of discrete categorical scores (k) and the y-axis shows the correlation between the observed categorical score (Y) and the underlying continuous score (u). The red horizontal line denotes a correlation of 0.95.

We first quantify the correlation between the observed categorical score ( *Y*) and the underlying continuous score ( *u*), as a function of the number of categories. Figure 2 shows the results from Equation [1], for k = 2 to 100. It shows there is very little loss of information when the number of categories is five or more. For example, the correlation is 0.958, 0.976, 0.987 and 0.992, for k = 5, 7, 10 and 13, respectively. The association between the correlation and the number of categories can be approximated by the simple equation, R(k) ≈ 1 – 0.7k ^-1.7^, which fits almost perfectly.

**Figure 3.**
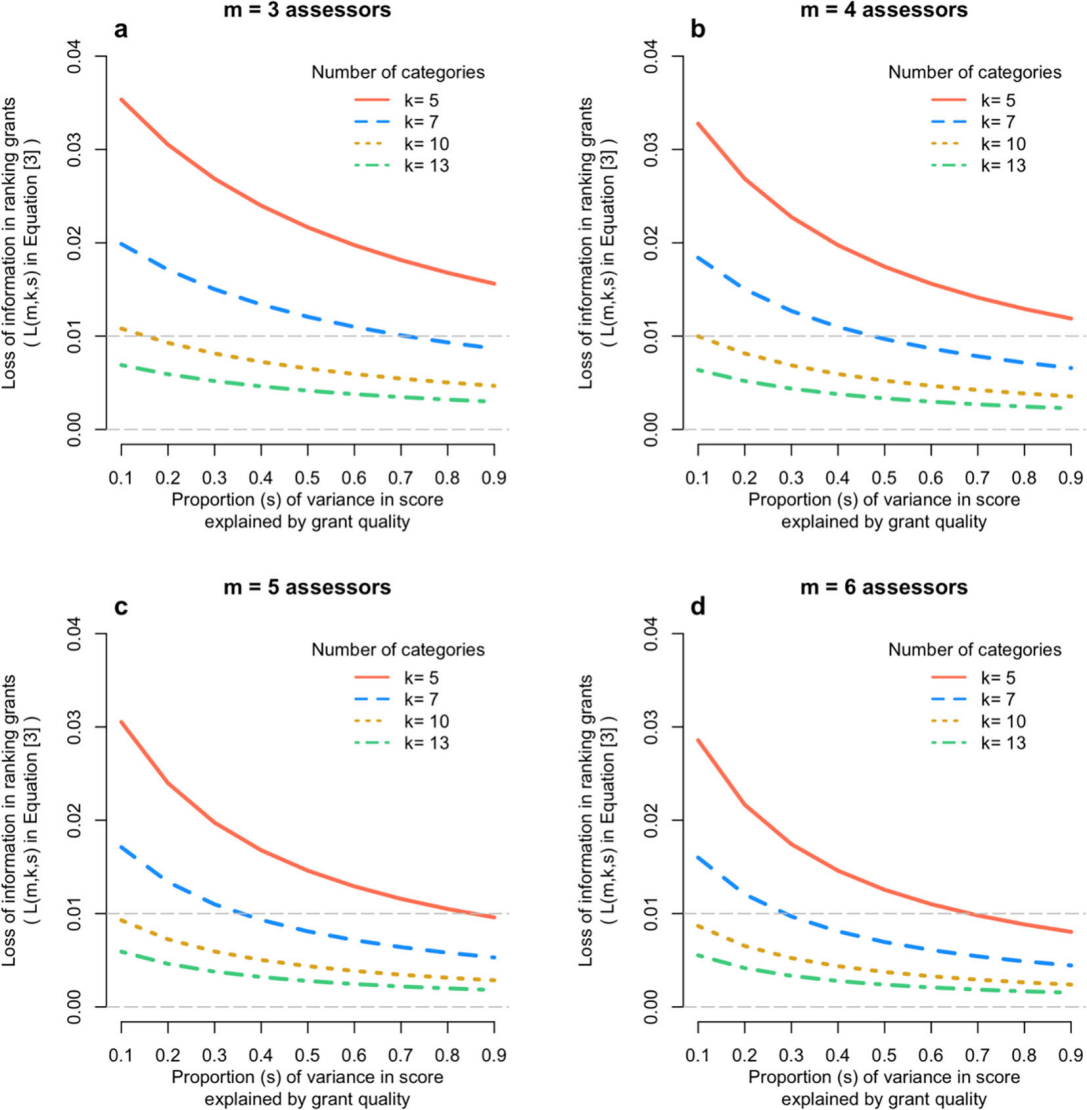
Loss of information relative to scoring on a continuous scale. Each panel shows the loss of information ( Equation [3]) when scoring a finite number of categories relative to the continuous score, as a function of the number of assessors (panels a to d) and the proportion of variation in scores due to the quality of the grant (x-axis).

Given the correlations in Figure 2 we calculated the correlation between the true quality of a grant ( *q*) and the mean score on the categorical scale from m assessors. Figure 3 shows the results from Equation [3], for m = 3,4,5,6; k = 5,7,10,13; and s from 0.1 to 0.9. It shows that that loss of information on the correlation between true quality of the grant and its mean assessor score is very small – typically 2% or less.

**Figure 4.**
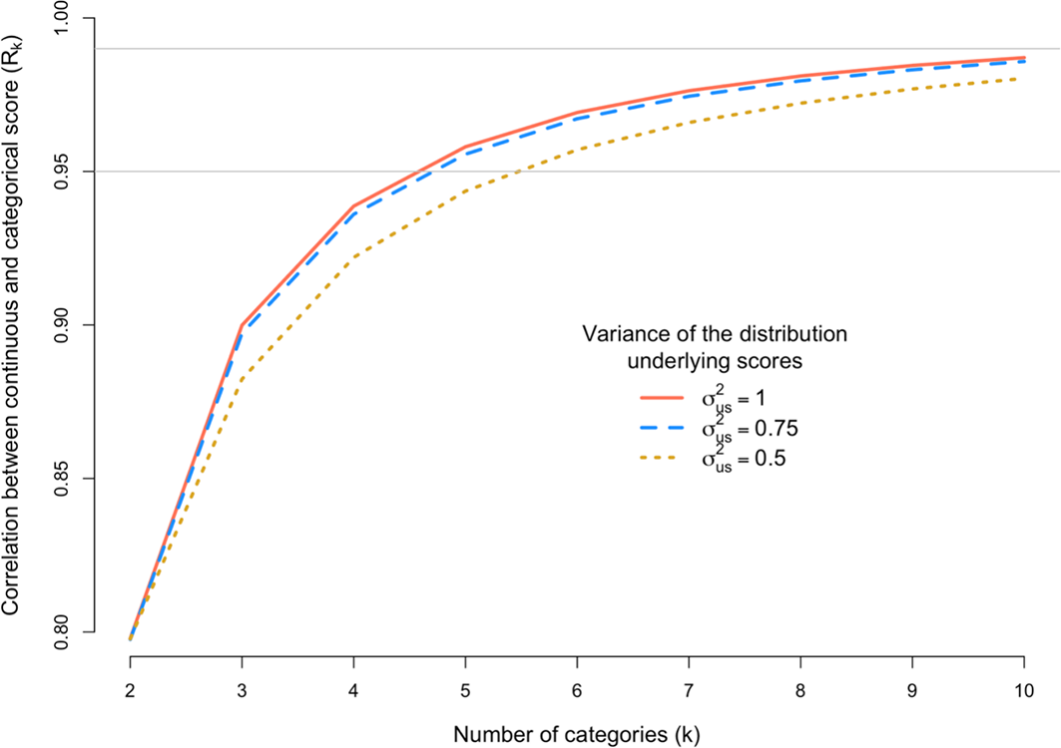
Loss of information induced by scoring too few grants in extreme categories. The x-axis is the number of discrete categorical scores (k) and the y-axis shows the correlation (R _k_) between the observed categorical score (Y) and the underlying continuous score (u). The correlation R _k_ is calculated under three scenarios defined by the variance (σ ^2^_s_) of the distribution of underlying scores. The grey horizontal line denotes a correlation of 0.95 or 0.99.

We next explored the scenario where grant assessors do not sufficiently use the entire scale available to them, by simulating σ ^2^_us_ < 1, which leads to a deficiency of scores in the tails of the distribution. For example, the proportion of scores for k = 5 in categories 1-5 ( Figure 1) are 10.3%, 23.4%, 32.6%, 23.4% and 10.3%, respectively, when the distribution underlying scores has a variance of σ ^2^_us_ = 1, but 3.7%, 23.9%, 44.8%, 23.9% and 3.7% when that variance is σ ^2^_us_ = 0.5. In this extreme scenario, the proportions in the tails are nearly 3-fold (10.3/3.7) lower than they should be yet decreasing σ ^2^_us_ from 1 to 0.5 induces only a small reduction of R _k_ from 0.958 to 0.944. Figure 4 shows R _k_ for a scoring scale with 2 to 10 categories when the variance of underlying distribution is σ ^2^_us_ = 0.5, 0.75 or 1.

## Discussion

Intuitively one might think that scoring with a broader scale is always better, but the results herein show that this can be misleading. Above k = 5 categories there is a very small gain in the signal-to-noise ratio compared to a fully continuous scale, and the effect on the accuracy of the ranking of grants is even smaller.

Comparing k = 5 with k = 10 categories and k = 7 with k = 13 categories shows a theoretical gain of 3% (0.987/0.958) and 1.6% (0.992/0.976) in the correlation between observed and continuous scales ( Figure 2). These very small gains predicted by doubling the number of categories scored will have to be balanced with the cost of changing the grant scoring systems.

The effect of ranking grants on their quality is even smaller. Figure 3 shows that, for most existing Australian grant scoring schemes, the loss in accuracy of scoring a grant using discrete categories compared to a truly continuous scale is trivial – nearly always less than 1%. As shown in the methods section, the squared correlation between the true quality of a grant and the average score from m assessors is m/(m + λ _Y_), with λ _Y_ = (1-R _k_^2^s)/(R _k_^2^s). Since R _k_^2^ is close to 1 ( Figure 2), the squared correlation is approximately equal to m/[m + (1-s)/s]. Therefore, even if the signal-to-noise ratio parameter s is as low as, say, 1/3, the squared correlation between the true quality and the mean assessor score is m/(m + 2), or 3/5, 2/3 and 5/7 for m = 3, 4 and 5, respectively, hence correlations ranging from 0.77 to 0.85.

The results in Figure 4 are to mimic a situation where assessors score too closely to the mean. As expected, R _k_ decreases when fewer grants are scored in the tails of the distribution of categories. However, the loss of information is generally very small. For example, for k = 7 and the most extreme case considered (σ ^2^_us_ = 0.5), R _k_ = 0.966, which is only slightly lower than 0.976, which is the correlation when the distribution of assessor scores is consistent with the underlying true distribution with variance of 1.

We have necessarily made a number of simplifying assumptions, but they could be relaxed in principle, for example different statistical distributions of the quality of the grant and the errors could be used. We have also assumed no systematic bias in scorers so that the true quality value of a grant on the observed scale is the mean value from a very large number of independent scorers. Departures from these assumptions will require additional assumptions and more parameters to model. However, assuming a multiple threshold model with normally distributed random effects on an underlying scale is simple and flexible and likely both robust and sufficient to address questions of the scale of grant scoring.

## Data availability

The data underlying Figures 1-3 are generated automatically by the provided R scripts.

## Software availability

Source code available from: https://github.com/loic-yengo/GrantSCoring_Figures

Archived source code at the time of publication: https://zenodo.org/record/7141342

License: Creative Commons Attribution 4.0 International license (CC-BY 4.0).
