# Supplementary material for: The effect of the scale of grant scoring on ranking accuracy
Source: F1000Res. 2023 Feb 6;11:1197. Originally published 2022 Oct 19. [Version 2] doi: 10.12688/f1000research.125400.2 (PMC9938341; doi:10.12688/f1000research.125400.2)
Supplement: Peer review history

# Peer review: Reviewer response for version 2

**DOI:** 10.5256/f1000research.143519.r162449

**Published:** 2023-02-17

**Authors:** Rachel Heyard

**Affiliations:** 1 Center for Reproducible Science and Department of Biostatistics at the Epidemiology, Biostatistics and Prevention Institute (EPBI), University of Zurich, Zurich, Switzerland

In their revision, the authors addressed all my comments. The introduction and motivation was extended, more details were added into the methods section and the discussion was adapted. 

 I have a couple of very minor comments that could, but do not have to be addressed: 

- p.4., end of first paragraph: change sentence "Note that in principle this model could be extended by adding a random effect for assessor (e.g., Erosheva et al., 2020), but that for our model derivations and results the variance due to assessor would also appears as noise." to "Note that in principle this model could be extended by adding a random effect for [the] assessor (e.g., Erosheva et al., 2020), but [in] our model derivations and results the variance due to [the] assessor would also appear[] as noise."
- Isn't z_i simply the  t_i-quantile of the standard normal distribution? 
- The "(results not shown)" at the end of the manuscript could probably be changed into a "(find analysis in R-script on github)". Meaning that since you performed the analysis, you could also push it to goit.
- The list in the github readme does not compile, making it hard to read.

Is the rationale for developing the new method (or application) clearly explained?

Partly

Is the description of the method technically sound?

Yes

Are the conclusions about the method and its performance adequately supported by the findings presented in the article?

Yes

If any results are presented, are all the source data underlying the results available to ensure full reproducibility?

Yes

Are sufficient details provided to allow replication of the method development and its use by others?

Partly

Reviewer Expertise:

Statistics, Grant peer review, Research evaluation

I confirm that I have read this submission and believe that I have an appropriate level of expertise to confirm that it is of an acceptable scientific standard.

# Peer review: Reviewer response for version 2

**DOI:** 10.5256/f1000research.143519.r162448

**Published:** 2023-02-16

**Authors:** Alejandra Recio-Saucedo

**Affiliations:** 1 National Institute for Health Research (NIHR) Collaboration for Applied Health Research and Care (CLAHRC) Wessex, University of Southampton, Southampton, UK

I would like to thank the authors for addressing the feedback in the revision process. Changes made to the article following the suggestions listed in the review have satisfactorily resolved all queries. 

 Findings and take away message provide important evidence to funding organisations when changes to application and funding processes are designed. Consideration of unintended consequences in terms of increased bureaucracy and cost when making changes to scoring scales is highly relevant in current research practice.

Is the rationale for developing the new method (or application) clearly explained?

Partly

Is the description of the method technically sound?

Yes

Are the conclusions about the method and its performance adequately supported by the findings presented in the article?

Yes

If any results are presented, are all the source data underlying the results available to ensure full reproducibility?

Yes

Are sufficient details provided to allow replication of the method development and its use by others?

Yes

Reviewer Expertise:

Research on Research; Health services delivery research

I confirm that I have read this submission and believe that I have an appropriate level of expertise to confirm that it is of an acceptable scientific standard.

# Peer review: Reviewer response for version 1

**DOI:** 10.5256/f1000research.137702.r157462

**Published:** 2022-12-21

**Authors:** Alejandra Recio-Saucedo

**Affiliations:** 1 National Institute for Health Research (NIHR) Collaboration for Applied Health Research and Care (CLAHRC) Wessex, University of Southampton, Southampton, UK

“ *The effect of the scale of grant scoring on ranking accuracy*” by Visscher and Yengo presents the work of a novel approach to understand the effect of scoring categories/scales (and assessors) on the loss of information/loss in accuracy of grant scoring. The authors developed a multiple threshold model with normally distributed random effects on an underlying scale and showed that doubling the number of categories used to score grant applications does not change the scoring accuracy (as defined by authors), highlighting the importance of assessing the cost of changing grant scoring systems against the benefit it adds to scoring. The authors also studied the correlation between observed categorical scores and continuous scores (as a function of the number of categories) reporting very little loss of information when the number of categories was five or more.

**Overall strengths:**

 Method and data availability: Providing the code to run the analysis reported in this paper is commendable. In doing this, the authors are supporting the principles of research openness and transparency that strengthens science and enhance its value to academia and society. It is a great example of the contribution of meta-research.

 Clarity of results: The authors provide a clear description of the main findings of their analysis in a succinct and informative way.

 Contribution from conclusions presented: In light of recent work ( *e.g., Independent Review of Research Bureaucracy Interim Report https://assets.publishing.service.gov.uk/government/uploads/system/uploads/attachment_data/file/1046070/independent-review-of-research-bureaucracy-interim-report.pdf*), the work presented is timely and highly relevant. Changes to the research funding system (from application to delivery and impact) need to be evidence-based. Showing that changes to scoring criteria in funding agencies are not necessarily the solution to issues linked to research funding decisions (which the authors do not necessarily discuss in their literature) is a clear contribution to efforts aimed at reducing unnecessary research bureaucracy.

**Areas for improvement:**

 The work described is not entirely linked to the title of the paper as there is no information on ranking accuracy except for a phrase towards the end of the discussion section: “…the true quality value of a grant on the observed scale is the mean value from a very large number of independent scorers.”

 They main area to address in this work is represented in the partial response to the question: Is the rationale for developing the new method clearly explained?

 Published work that reports studies exploring variation in scores touches on one of the issues that the authors are studying, i.e., what is the right number of assessors to ensure a research application is scored fairly/bias-free (through less score variability), whilst ensuring the process is efficient and viable (please see *Kaplan D, Lacetera N, Kaplan C. Sample size and precision in NIH peer review. PLoS One. 2008;3(7):e2761. https://doi.org/10.1371/journal.pone.0002761.*) This, and other related work on scores inter-reliability (e *.g., Sattler DN, McKnight PE, Naney L, Mathis R. Grant peer review: improving inter-rater reliability with training. PLoS One. 2015;10(6):e0130450. https://doi.org/10.1371/journal.pone.0130450.S*) highlight the aspect of this paper that is not presented about how this work is contributing to the efforts of enhancing decision-making in research funding. The key assumption of the authors’ model of no-systematic bias in scores provides a ‘place to start’, but the need to include variability because in reality those scenarios are more likely to be the norm leaves a gap in the contribution of the work reported.

 The model proposed successfully shows that all things being equal, having 13 scoring categories does not enhance an application score, making a case for simplified scoring criteria that have the potential of reducing administrative burden. However, the value of the work will increase if the model showed similar results in the presence of systematic bias in scores.

 Overall, the paper needs to provide context to how their work contributes to the issues that have been identified with research funding allocation for which innovative alternatives have been suggested (see for example *Guthrie S, Ghiga I, Wooding S. What do we know about grant peer review in the health sciences? [version 2; peer review: 2 approved]. F1000Research. 2018;6:1335; Guthrie S, Guérin B, Wu H, Ismail S, Wooding S. Alternatives to peer review in research project funding: 2013 Update. Santa Monica: RAND Corporation; 2013. https://www.rand.org/pubs/research_reports/RR139.html*.) The results reported are promising, however, without reference to the context and processes that are being targeted to improve, the value of the work is not as evident as it could be.

Is the rationale for developing the new method (or application) clearly explained?

Partly

Is the description of the method technically sound?

Yes

Are the conclusions about the method and its performance adequately supported by the findings presented in the article?

Yes

If any results are presented, are all the source data underlying the results available to ensure full reproducibility?

Yes

Are sufficient details provided to allow replication of the method development and its use by others?

Yes

Reviewer Expertise:

Research on Research; Health services delivery research

I confirm that I have read this submission and believe that I have an appropriate level of expertise to confirm that it is of an acceptable scientific standard, however I have significant reservations, as outlined above.

## References

[1] : Sample size and precision in NIH peer review. *PLoS One* .2008;3(7) : 10.1371/journal.pone.0002761 e2761 10.1371/journal.pone.0002761 18648494PMC2447157

[2] : Grant Peer Review: Improving Inter-Rater Reliability with Training. *PLoS One* .2015;10(6) : 10.1371/journal.pone.0130450 e0130450 10.1371/journal.pone.0130450 26075884PMC4468126

[3] : What do we know about grant peer review in the health sciences?. *F1000Res* .2017;6: 10.12688/f1000research.11917.2 1335 10.12688/f1000research.11917.2 29707193PMC5883382

[4] : Alternatives to peer review in research project funding. *Santa Monica: RAND Corporation* .2013; Reference source

# Response

**Published:** 2023-01-22

**Authors:** Peter Visscher

**Affiliations:** Queensland Brain Institute, The University of Queensland, Australia

“The effect of the scale of grant scoring on ranking accuracy” by Visscher and Yengo presents the work of a novel approach to understand the effect of scoring categories/scales (and assessors) on the loss of information/loss in accuracy of grant scoring. The authors developed a multiple threshold model with normally distributed random effects on an underlying scale and showed that doubling the number of categories used to score grant applications does not change the scoring accuracy (as defined by authors), highlighting the importance of assessing the cost of changing grant scoring systems against the benefit it adds to scoring. The authors also studied the correlation between observed categorical scores and continuous scores (as a function of the number of categories) reporting very little loss of information when the number of categories was five or more.

**We thank the reviewer for their time, comments and suggestions. We thank the reviewer in particular for pointing us to a number of relevant publications which was most helpful because peer review research is not our normal area of research. We have revised the manuscript taking those comments and suggestions into account.**

 Overall strengths:

 Method and data availability: Providing the code to run the analysis reported in this paper is commendable. In doing this, the authors are supporting the principles of research openness and transparency that strengthens science and enhance its value to academia and society. It is a great example of the contribution of meta-research.

 Clarity of results: The authors provide a clear description of the main findings of their analysis in a succinct and informative way.

 Contribution from conclusions presented: In light of recent work (e.g., Independent Review of Research Bureaucracy Interim Report https://assets.publishing.service.gov.uk/government/uploads/system/uploads/attachment_data/file/1046070/independent-review-of-research-bureaucracy-interim-report.pdf), the work presented is timely and highly relevant. Changes to the research funding system (from application to delivery and impact) need to be evidence-based. Showing that changes to scoring criteria in funding agencies are not necessarily the solution to issues linked to research funding decisions (which the authors do not necessarily discuss in their literature) is a clear contribution to efforts aimed at reducing unnecessary research bureaucracy.

 Areas for improvement:

 The work described is not entirely linked to the title of the paper as there is no information on ranking accuracy except for a phrase towards the end of the discussion section: “…the true quality value of a grant on the observed scale is the mean value from a very large number of independent scorers.”

 They main area to address in this work is represented in the partial response to the question: Is the rationale for developing the new method clearly explained?

 Published work that reports studies exploring variation in scores touches on one of the issues that the authors are studying, i.e., what is the right number of assessors to ensure a research application is scored fairly/bias-free (through less score variability), whilst ensuring the process is efficient and viable (please see Kaplan D, Lacetera N, Kaplan C. Sample size and precision in NIH peer review. PLoS One. 2008;3(7):e2761. https://doi.org/10.1371/journal.pone.0002761.) This, and other related work on scores inter-reliability (e.g., Sattler DN, McKnight PE, Naney L, Mathis R. Grant peer review: improving inter-rater reliability with training. PLoS One. 2015;10(6):e0130450. https://doi.org/10.1371/journal.pone.0130450.S) highlight the aspect of this paper that is not presented about how this work is contributing to the efforts of enhancing decision-making in research funding. The key assumption of the authors’ model of no-systematic bias in scores provides a ‘place to start’, but the need to include variability because in reality those scenarios are more likely to be the norm leaves a gap in the contribution of the work reported.

**We thank the reviewer for these references which we have now cited. We have expended on our Introduction and Discussion sections to place our study in the peer review literature. We also give our motivation for the study.**

 The model proposed successfully shows that all things being equal, having 13 scoring categories does not enhance an application score, making a case for simplified scoring criteria that have the potential of reducing administrative burden. However, the value of the work will increase if the model showed similar results in the presence of systematic bias in scores.

**We agree with the reviewer that we have focused on precision only and not on bias. The reason is the narrow scope of our paper: what happens if, everything else being the same, the scale of scoring changes? As we now mention, our results are also relevant if there is a random effect of assessor, such that some assessors score on average lower or higher than others. Our motivation for focussing on precision is that there is strong evidence in the literature of lack of precision yet to our knowledge less evidence for systematic biases (e.g., Guthrie et al. 2018). To study systematic bias would require us to model such biases and there are many ways in which this could be done and the necessary assumptions we would need to make could of course be questioned. A full exploration of a wide range of models for bias will in our opinion require extensive computer simulations and we judged that to be outside of the scope of our study.**

 Overall, the paper needs to provide context to how their work contributes to the issues that have been identified with research funding allocation for which innovative alternatives have been suggested (see for example Guthrie S, Ghiga I, Wooding S. What do we know about grant peer review in the health sciences? [version 2; peer review: 2 approved]. F1000Research. 2018;6:1335; Guthrie S, Guérin B, Wu H, Ismail S, Wooding S. Alternatives to peer review in research project funding: 2013 Update. Santa Monica: RAND Corporation; 2013. https://www.rand.org/pubs/research_reports/RR139.html.) The results reported are promising, however, without reference to the context and processes that are being targeted to improve, the value of the work is not as evident as it could be.

 Is the rationale for developing the new method (or application) clearly explained?

**We thank the reviewer again for providing these references which we have now cited.**

# Peer review: Reviewer response for version 1

**DOI:** 10.5256/f1000research.137702.r153690

**Published:** 2022-11-07

**Authors:** Rachel Heyard

**Affiliations:** 1 Center for Reproducible Science and Department of Biostatistics at the Epidemiology, Biostatistics and Prevention Institute (EPBI), University of Zurich, Zurich, Switzerland

The paper by Peter Visscher and Loic Yengo addresses a crucial question for funding bodies: how large is the effect of the scoring scale on the proposal ranking accuracy. The manuscript is concise and well-written. R-code is available on github and computationally reproducible, which is wonderful! My main concern with the paper as it is currently presented, is that it is not well integrated in the literature on grant peer review (so far only three papers from Genetics, all from before 1990, are cited).

**Introduction**:

- The introduction is a bit short and the whole paper has only three references, which are also very old. There has been a lot of research on the reliability and accuracy of grant peer review scores which the authors could at least mention.  
- Funding decisions are based on the rankings of grant proposals. Therefore the accuracy of the ranking of grant proposals is so important. For a general audience, less familiar with grant peer review, some more insights into the motivation of the work could be relevant. 

**Methods**:

- I applaud the authors for uploading their R-code on github, which helped me a lot in understanding of the methods. From the methods section in the paper itself it was rather hard to grasp all the details. Therefore reconsider reworking the methods section and give more details: e.g. how are p _i_ and z _i_ computed? 
- The ordering of the presentation of the methods could be changed to make the section more reader-friendly: for example, does the third paragraph start with explaining how the thresholds can be computed by maximising the correlation between Y and u while this correlation is only defined at the very end of the paragraph when explaining the loss of information problem.
- The multiple threshold model is set in a way that most weight is on the average score (3 for a 5-point scale). From the literature on grant peer review we know however that the actual distributions are most often skewed towards higher scores (see for example the plots in doi:10.1126/sciadv.aaz4868 ^1^). Can your multiple threshold model be updated to such a more realistic scenario?
- Often in grant peer review the same experts might score more than one proposal which will affect the error structure of your normal distributions, as all scores by expert xyz are correlated with each other. Could such a scenario be included in your multiple threshold model?

 

**Discussion**: 

- I believe the discussion is also lacking more context. What do your results mean for funding decisions? for funding agencies?
- The thresholds of the model are chosen in a way to maximise the correlation between the observed and the continuous scale. How realistic is this? Isn't it this exact subjective choice made by the reviewers which often makes grant peer review unreliable? If a 4 in a 5-point scale means the same for all reviewers (as it is the case in your model, right?), grant peer review would be almost perfectly reliable. However it is a very subjective choice. Can you comment on this? at least as a limitation in the discussion.

**General Comment**: 

- The title suggests that you are interested in ranking of grant proposals, but in the article you are actually interested in the correlations between the scoring and the true quality (!= not a rank) of the proposals. The relationship between the effects on the scoring and the subsequent ranks of proposals should be explained more clearly.

**Code**:

- All four R-scripts for the four figures re-define the function 'CorrEquiSpaced'. It is common practice to instead write an R-script (called functions.R) with all the functions and very detailed description. All scripts could benefit from more comments, also the Readme file is not very informative. 

Is the rationale for developing the new method (or application) clearly explained?

Partly

Is the description of the method technically sound?

Yes

Are the conclusions about the method and its performance adequately supported by the findings presented in the article?

Yes

If any results are presented, are all the source data underlying the results available to ensure full reproducibility?

Yes

Are sufficient details provided to allow replication of the method development and its use by others?

Partly

Reviewer Expertise:

Statistics, Grant peer review, Research evaluation

I confirm that I have read this submission and believe that I have an appropriate level of expertise to confirm that it is of an acceptable scientific standard, however I have significant reservations, as outlined above.

## References

[1] : NIH peer review: Criterion scores completely account for racial disparities in overall impact scores. *Sci Adv* .6(23) : 10.1126/sciadv.aaz4868 eaaz4868 10.1126/sciadv.aaz4868 32537494PMC7269672

# Response

**Published:** 2023-01-22

**Authors:** Peter Visscher

**Affiliations:** Queensland Brain Institute, The University of Queensland, Australia

The paper by Peter Visscher and Loic Yengo addresses a crucial question for funding bodies: how large is the effect of the scoring scale on the proposal ranking accuracy. The manuscript is concise and well-written. R-code is available on github and computationally reproducible, which is wonderful! My main concern with the paper as it is currently presented, is that it is not well integrated in the literature on grant peer review (so far only three papers from Genetics, all from before 1990, are cited).

**We thank the reviewer for their time, comments and suggestions. We thank the reviewer in particular for pointing us to a number of relevant publications which was most helpful because peer review research is not our normal area of research. We have revised the manuscript taking those comments and suggestions into account.**

 Introduction:

 The introduction is a bit short and the whole paper has only three references, which are also very old. There has been a lot of research on the reliability and accuracy of grant peer review scores which the authors could at least mention. 

**We thank the reviewer for this comment and have now expanded the Introduction and have cited a number of relevant papers and reports.**

 Funding decisions are based on the rankings of grant proposals. Therefore the accuracy of the ranking of grant proposals is so important. For a general audience, less familiar with grant peer review, some more insights into the motivation of the work could be relevant.

**We have now included a statement about the motivation.**

 Methods:

 I applaud the authors for uploading their R-code on github, which helped me a lot in understanding of the methods. From the methods section in the paper itself it was rather hard to grasp all the details. Therefore reconsider reworking the methods section and give more details: e.g. how are pi and zi computed?

**We have tweaked the Methods section and have specified how, in R, pi and zi are computed.**

 The ordering of the presentation of the methods could be changed to make the section more reader-friendly: for example, does the third paragraph start with explaining how the thresholds can be computed by maximising the correlation between Y and u while this correlation is only defined at the very end of the paragraph when explaining the loss of information problem.

**We thank the referee for this comment. We have now added a few sentences for clarification and define the correlation earlier.**

 The multiple threshold model is set in a way that most weight is on the average score (3 for a 5-point scale). From the literature on grant peer review we know however that the actual distributions are most often skewed towards higher scores (see for example the plots in doi:10.1126/sciadv.aaz48681). Can your multiple threshold model be updated to such a more realistic scenario?

**We thank the referee for this comment. We note that the distribution of the Overall Impact Score in Erosheva et al. 2020 (Fig2), on which the ranking is based, is much less skewed than the individual component scores and furthermore that the distribution is a mixture because two groups of grants are compared. The distribution of scores when adjusted for the fixed effect may be (even) more normal. In any case, as we mention the Discussion, in principle the effect of the scale can be investigated with other statistical distributions, for example assuming that the continuous scale is log-normal (skewed) or kurtotic. Non-normal distributions of the quality and error term will make the results less generalisable because the of thresholds will depend on the specific parameters of the chosen non-normal distribution. For example, Gianola (1979) showed that for an exponential distribution, the threshold values depend on the densities of q and e, and therefore each signal-to-noise ratio will have its own set of thresholds. A full exploration of a wide range of statistical models will in our opinion require extensive computer simulations and we judged that to be outside of the scope of our study.**

 Often in grant peer review the same experts might score more than one proposal which will affect the error structure of your normal distributions, as all scores by expert xyz are correlated with each other. Could such a scenario be included in your multiple threshold model?

**We thank the referee for this question. For our theoretical calculations, the variance due to assessor (as a random effect) is also noise and we have clarified this in the revision. For real data analysis we agree with the reviewer that an assessor effect should be fitted (as, for example, done in Erosheva et al., Science Advances 2020), if only to quantify how much variation in scores is due to some assessors scoring systematically lower or higher than others.**

 Discussion:

 I believe the discussion is also lacking more context. What do your results mean for funding decisions? for funding agencies?

**We have now expanded the Discussion significantly.**

 The thresholds of the model are chosen in a way to maximise the correlation between the observed and the continuous scale. How realistic is this? Isn't it this exact subjective choice made by the reviewers which often makes grant peer review unreliable? If a 4 in a 5-point scale means the same for all reviewers (as it is the case in your model, right?), grant peer review would be almost perfectly reliable. However it is a very subjective choice. Can you comment on this? at least as a limitation in the discussion.

**We thank the reviewer for this question. We agree that in the absence of measurement error (or subjective choices) the reliability of scoring would be perfect, but there is strong evidence that assessors do not always score the same even if the category descriptors are well-defined. We give examples of why assessors might score differently in the revised manuscript. The choice of our thresholds was just to make a link between a true continuous scale and the actual scale of scoring and this choice seems reasonable to us. The main assumption is that the true distribution is continuous.**

 General Comment:

 The title suggests that you are interested in ranking of grant proposals, but in the article you are actually interested in the correlations between the scoring and the true quality (!= not a rank) of the proposals. The relationship between the effects on the scoring and the subsequent ranks of proposals should be explained more clearly.

**We thank the reviewer for this point. We have now specified that our parameters are directly related to single-rater reliability and reliability based upon the mean from multiple assessors. In addition, from our simulations we compared the Spearman rank correlations to the Pearson correlations. The Spearman correlations were even larger than the Pearson correlations (Figure for referee below) and therefore re-enforce the conclusion of our study. We have added a paragraph in the Discussion: “Throughout this study we have used the Pearson correlation to quantify the correlation between the score on the underlying and observed scales. We could also have used the Spearman rank correlation, but the conclusions would not change. In fact, the Spearman rank correlations are even larger than the Pearson correlations and they converge at k = 10 categories (results not shown).”**

 See graph here: https://f1000research.s3.amazonaws.com/linked/481471.Visscher_1.png

 Code:

 All four R-scripts for the four figures re-define the function 'CorrEquiSpaced'. It is common practice to instead write an R-script (called functions.R) with all the functions and very detailed description. All scripts could benefit from more comments, also the Readme file is not very informative.

**We thank the reviewer for this comment and have now updated our R script accordingly, added more comments to the code and expanded the Readme file. The github link remains unchanged but the updated version of the Zenodo link is available at** https://doi.org/10.5281/zenodo.7519164
